# Reactivity of Shale to Supercritical CO_2_: Insights from Microstructural Characterization and Mineral Phase Evolution in Caney Shales for CCUS Applications

**DOI:** 10.3390/ma18143382

**Published:** 2025-07-18

**Authors:** Loic Bethel Dje, Mileva Radonjic

**Affiliations:** Barrier Materials and Geomimicry Lab, School of Chemical Engineering, Oklahoma State University, Stillwater, OK 74078, USA

**Keywords:** geochemical CO_2_ sequestration, supercritical CO_2_, mineral trapping in shale

## Abstract

Understanding mineral–fluid interactions in shale under supercritical CO_2_ (scCO_2_) conditions is relevant for assessing long-term geochemical containment. This study characterizes mineralogical transformations and elemental redistribution in five Caney Shale samples serving as proxies for reservoir (R1, R2, R3) and caprock (D1, D2) facies, subjected to 30-day static exposure to pure scCO_2_ at 60 °C and 17.23 MPa (2500 psi), with no brine or impurities introduced. SEM-EDS analyses were conducted before and after exposure, with mineral phases classified into silicates, carbonates, sulfides, and organic matter. Initial compositions were dominated by quartz (38–47 wt.%), illite (16–23 wt.%), carbonates (12–18 wt.%), and organic matter (8–11 wt.%). Post-exposure, carbonate loss ranged from 15 to 40% in reservoir samples and up to 20% in caprock samples. Illite and K-feldspar showed depletion of Fe^2+^, Mg^2+^, and K^+^ at grain edges and cleavages, while pyrite underwent oxidation with Fe redistribution. Organic matter exhibited scCO_2_-induced surface alteration and apparent sorption effects, most pronounced in R2 and R3. Elemental mapping revealed Ca^2+^, Mg^2+^, Fe^2+^, and Si^4+^ mobilization near reactive interfaces, though no secondary mineral precipitates formed. Reservoir samples developed localized porosity, whereas caprock samples retained more structural clay integrity. The results advance understanding of mineral reactivity and elemental fluxes in shale-based CO_2_ sequestration.

## 1. Introduction

Geologic carbon sequestration (CCS) is a leading strategy for reducing atmospheric CO_2_ levels through long-term storage in deep subsurface formations [1]. Shale formations, commonly utilized as caprocks, are now also being explored as storage media due to their fine-grained texture, high clay content, and nanoporous structure [2]. These characteristics enhance surface reactivity and support mechanisms such as sorptive trapping and geochemical immobilization.

Exposure of shale to supercritical CO_2_ (scCO_2_) can initiate a range of mineral–fluid interactions. Carbonate phases may undergo dissolution; phyllosilicates, particularly illite, can experience elemental leaching and structural disruption; and sulfides like pyrite may oxidize. These transformations can alter porosity, permeability, and long-term seal integrity [3,4]. Clay minerals are especially chemically reactive due to their high surface area and exchangeable interlayer cations, which contribute to buffering and ion transport under scCO_2_ conditions [5,6]. Additionally, scCO_2_ has a higher diffusivity than water, allowing it to access mineral surfaces more efficiently and drive reactions within micro/nanopores and microfractures [7].

**Figure 1:**
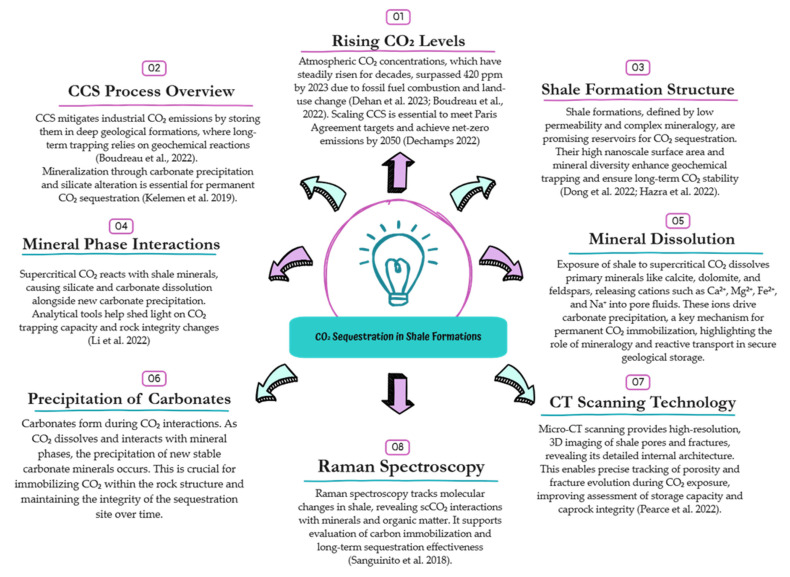
Summarized context of the research and the key questions this study covers [5,9,10,11,12,13,14,15,16].

Previous studies have frequently incorporated brine or gas impurities, making it difficult to isolate the effects of CO_2_ alone. Mineral transformations occur in shales under scCO_2_–SO_2_ conditions [8], but the independent behavior of shale minerals in the presence of pure scCO_2_ remains poorly understood. Moreover, comparative data on reservoir and caprock lithofacies from the same shale formation under identical conditions are limited. Figure 1 illustrates the various study gaps and our comprehensive experimental ideology.

This study addresses that gap by examining mineralogical and geochemical changes in reservoir- and caprock-proxy samples from the Caney Shale after static exposure to pure scCO_2_ at 60 °C and 17.23 MPa (2500 psia) for 30 days. Pre- and post-exposure characterization was performed using scanning electron microscopy with energy-dispersive X-ray spectroscopy (SEM-EDS), computed tomography (CT), and RAMAN spectroscopy to assess mineral phase changes, elemental redistribution, and structural modifications. The objective is to determine the following: (1) which mineral phases exhibit early-stage reactivity under pure scCO_2_ conditions; (2) how reactivity differs between reservoir and caprock proxies; and (3) what microstructural features indicate the onset of mineral trapping or porosity evolution. By integrating SEM-EDS, CT, and RAMAN spectroscopy with systematic phase classification across distinct lithofacies, this study establishes a baseline for understanding the intrinsic reactivity of shale formations under pure CO_2_ conditions. The next section outlines the experimental setup, sample selection, and analytical procedures used to evaluate these transformations.

Insights from the literature review account for various research aspects, which this study will seek to answer. Table 1 provides a summary of the comprehensive literature.

## 2. Materials and Methods

**Figure 2:**
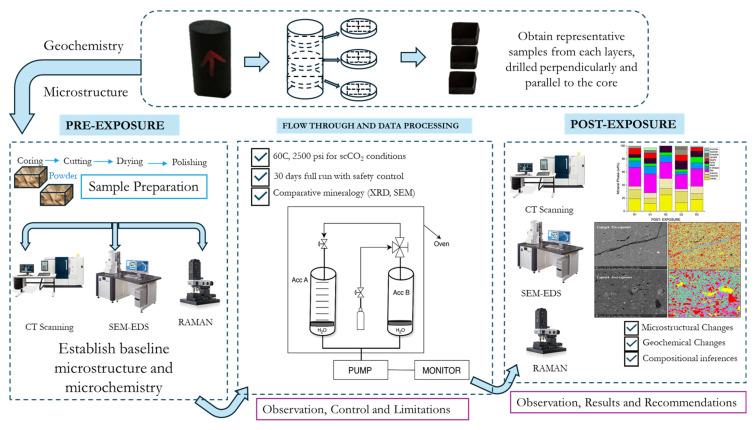
Comprehensive methodology. Accumulator (Acc.) B is filled with CO_2_ and set to the supercritical conditions of 35 °C and 8.27 MPa, and increased progressive in steps of 0.7 MPa and 5 °C to reaction conditions (60 °C and 17.2 MPa). This is performed with Acc. A closed. Accumulator A contains the shelve on which the samples are stacked on trays, avoiding any contamination. Pressure is maintained using an Isco dual piston pump.

Shale samples were sourced from the Caney Formation found within the South-Central Oklahoma Oil Province (SCOOP) in the Ardmore basin, characterized by fine-grained, organic-rich lithofacies. Two distinct zones were selected to represent reservoir and caprock analogs: Reservoir-proxy samples (R1, R2, R3) originated from the brittle zone and Caprock-proxy samples (D1, D2) came from the ductile zone. Figure 2 shows our experimental flow.

Samples were cut and polished into 1 cm × 1 cm × 0.5 cm cubical for exposure to scCO_2_. For each sample, two orientations were obtained: one parallel and one perpendicular to the bedding plane. This approach captured anisotropic responses to scCO_2_ exposure. Each analyzed surface was further segmented into four quadrants and a central point (Point 5) to account for heterogeneity during post-exposure analysis.

**Figure 3:**
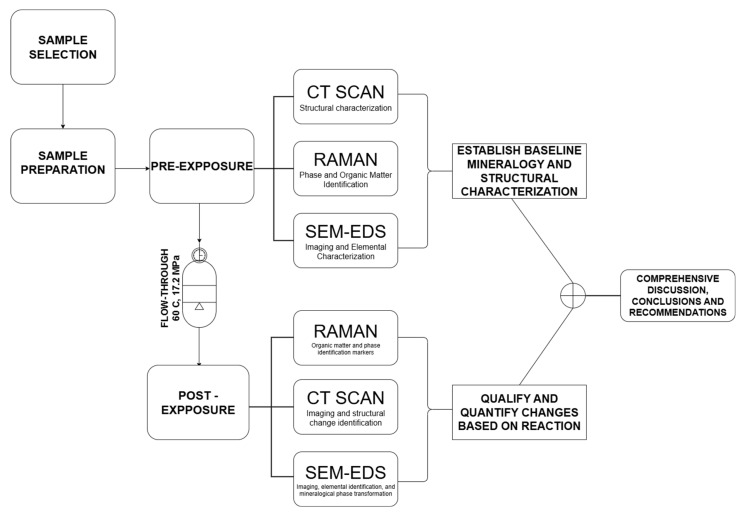
Experimental flowchart, displaying the thought-process and symbiotic use of microscopy and spectroscopy for phase identification and the effort to achieve scientific experimental results convergence towards understanding reaction mechanisms and their potential field consequences.

Pre and Post-exposure characterization involved a combination of imaging and spectroscopic methods to evaluate structural and chemical alterations (Figure 3). Unless otherwise stated, the equipment used are located at the Barrier Materials and Geomimicry lab. Due to the scale of interpretation, the CT scan, using Yxlon FF20 CT High-resolution (Comet Yxlon, Hamburg, Germany) was not adequate for this experiment and next experiments we plan to employ nano-CT. Samples were analyzed using scanning electron microscopy with energy-dispersive X-ray spectroscopy (SEM-EDS) using SEM imaging was carried out using a SCIOS Scanning Electron Microscope located at the OSU microscopy laboratory (Columbus, OH, USA). Quadrant-based mapping and a central point (Point 5) were examined at magnifications ranging from 500× to 5000×. Phases were classified as silicates (quartz, illite, feldspar), carbonates (calcite, dolomite), sulfides (pyrite), and organic matter. Elemental maps were used to track Fe, Mg, Ca, Si, K, and Al redistribution. Raman analysis was performed using an Alpha 300 R Raman Imaging Microscope (WITec GmbH, Ulm, Germany) over a 1 × 1 cm area, with spatial resolution of approximately 1 µm. Both 532 nm and 785 nm excitation lasers were employed. Spectra were acquired using 20× and 50× objective lenses, with the 532 nm laser dispersed by a 600 g/mm grating (BLZ = 500 nm). Laser power ranged from 0.5 to 5 mW, and the integration time was set to 1 s. This method identified shifts in carbonate peak positions, disordering in clay mineral structures, and potential alterations in organic matter. Spectra were collected at 20× magnification to support correlation with SEM-EDS data.

## 3. Results

Post-exposure SEM-EDS analyses revealed distinct mineralogical changes across reservoir and caprock facies, driven by phase-specific reactivity to scCO_2_. This section details the identified mineral phases, their compositional shifts, and morphological alterations after 30-day exposure.

### 3.1. Mineral Phase Identification

**Figure 4:**
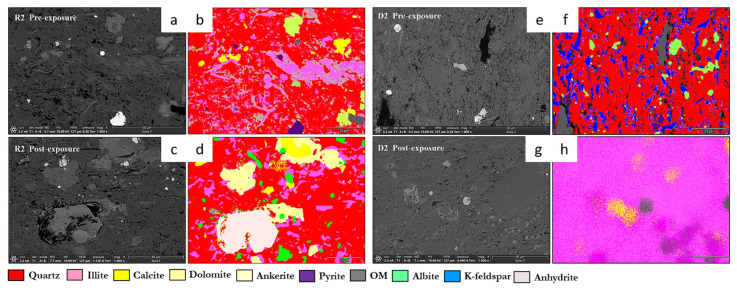
Mineral phase variability between reservoir and caprock proxies shown through SEM and mineral phase maps of R2 (**a**–**d**) and D2 (**e**–**h**) before and after scCO_2_ exposure, illustrating facies-specific mineralogical transformations and implications for CO_2_ sequestration. R2 (**a**–**d**)—The reservoir facies exhibit significant mineral reactivity, with pre-exposure assemblages of quartz, illite, anhydrite, albite, calcite, and ankerite transitioning to a more reactive post-exposure system marked by secondary dolomite, kaolinite, pyrite, and abundant organic matter. Widespread feldspar and carbonate dissolution, coupled with illite compositional changes, reflect active fluid–mineral interactions enhancing both porosity and mineral trapping potential. D2 (**e**–**h**)—In contrast, the caprock facies remains comparatively stable. While pre-exposure composition includes OM, illite (K and Na), and ankerite, post-exposure changes are more subdued, characterized by moderate growth in kaolinite and dolomite, persistence of illite, and limited carbonate transformation. This mineralogical resilience, dominated by low-permeability clays, supports long-term capillary sealing and geochemical buffering under dry scCO_2_ conditions.

**Figure 5:**
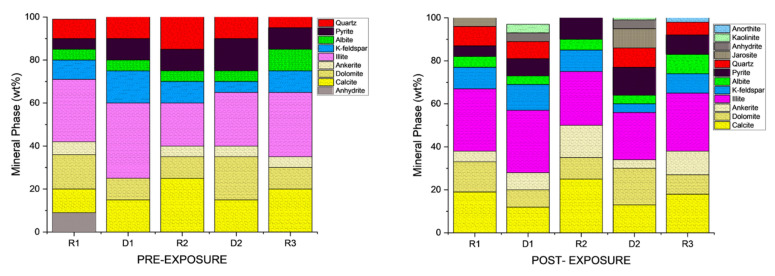
Bulk mineralogical compositions pre and post CO_2_ exposure for the reservoir and caprock proxies, based on EDS results and compared with x-ray diffraction. In reservoirs, scCO_2_ induced clear transformations: organic matter increased substantially (R2: 6.57 to 12.77 wt.%), while calcite and ankerite declined (R2 calcite: −1.21 wt.%, ankerite: −1.16 wt.%). New phases such as dolomite, jarosite, and wollastonite emerged, indicating active carbonate alteration and secondary mineral formation. Quartz and illite remained relatively stable. In caprock proxies, changes were more restrained. Organic matter still rose sharply (D2: 6.99 to 14.19 wt.%), with minor reductions in calcite and pyrite, and the appearance of jarosite and calcium sulfate. Key clays (illite, kaolinite) and framework silicates showed minimal change, reflecting strong geochemical variations.

Qualitative and morphological analyses from SEM-EDS revealed clear mineralogical heterogeneity across the Caney Shale facies. Samples were mapped at five locations per face four quadrants and a central point to ensure spatial representativity. SEM-backscattered imaging and EDS revealed distinct phase compositions and early-stage alterations across the Caney Shale samples after 30 days of pure scCO_2_ exposure. Reservoir and caprock proxies showed both compositional heterogeneity and phase-specific responses following scCO_2_ exposure. All five samples exhibited increases in organic matter (OM) visibility, while silicate and sulfate phases developed locally under reactive conditions. Primary mineral phases identified include quartz, illite, calcite, dolomite, K-feldspar, pyrite, and organic matter as shown in Figure 4, with a cross mineralogical comparison in Figure 5. Phase names followed standard SEM-EDS conventions. Where polymorphs were possible, identification was guided by morphology and elemental context; ambiguous cases used broader terms with noted limitations.

- Quartz remained the principal framework silicate across all facies. In reservoir proxies, pre-exposure abundances were 43.77 wt.% (R1), 42.23 wt.% (R2), and 45.96 wt.% (R3), with post-exposure values of 43.94 wt.%, 42.22 wt.%, and 45.68 wt.%, respectively. In caprock proxies, quartz accounted for 33.79 wt.% (D1) and 33.69 wt.% (D2) prior to exposure, increasing marginally to 34.13 wt.% (D1) and decreasing to 32.75 wt.% (D2) post-exposure. Across all facies, quartz grains retained angular, sharp morphologies with no significantly observable structural or chemical alteration.
- K-Feldspar (KAlSi_3_O_8_). K-feldspar was consistently present in all samples. In reservoirs, values ranged from 5.06 to 5.84 wt.% pre-exposure and from 5.89 to 5.99 wt.% post-exposure. In D1 and D2, K-feldspar were 7.06 wt.% and 6.54 wt.% pre-exposure, increasing to 7.13 wt.% in D1 and decreasing mildly to 6.36 wt.% for D2 post-exposure. No dissolution or surface roughening was evident under SEM imaging.
- Illite [(K,H_3_O)(Al,Mg,Fe)_2_(Si,Al)_4_O_10_(OH)_2_]. Illite occurred in all facies and was typically distributed along grain boundaries or within clay-rich matrices. Illite content in the reservoir proxies increased from 17.69 to 17.61 wt.% (R3) and 18.40 to 18.67 wt.% (R2). In caprock proxies, illite content increased from 17.26 to 17.47 wt.% (D1) and 16.31 to 16.68 wt.% (D2); pre and postexposure. Platy textures remained intact, although localized thinning and roughening of particle edges were noted in R2 and R3.
- Kaolinite (Al_2_Si_2_O_5_(OH)_4_). Kaolinite was identified in D1 and D2. Its abundance increased from 10.03 to 10.13 wt.% (D1) and decreased from 9.89 to 9.76 wt.% (D2); pre and post-exposure, respectively. Kaolinite maintained blocky morphology with no signs of chemical erosion or micro-pitting.
- Paragonite (NaAl_2_(Si_3_Al)O_10_(OH)_2_). Paragonite was not detected in any sample prior to exposure. Post-exposure, it appeared in R2 (0.38 wt.%), R3 (0.88 wt.%), D1 (0.72 wt.%), and D2 (0.90 wt.%). It was typically observed near altered illite flakes and within fine-grained matrix zones, forming as secondary Na-bearing phyllosilicate lamella.
- Calcite (CaCO_3_). Calcite was present in all samples, particularly in the reservoir facies. Calcite decreased slightly from 9.92 to 9.01 wt.% (R1), 9.97 to 8.76 wt.%, and increased from 8.80 to 9.05 wt.% (R3). In caprocks, it increased from 9.41 to 9.75 wt.% (D1) and decreased from 9.62 to 8.80 wt.% (D2). SEM images revealed surface pitting and edge retreat, especially in R1 and R2.
- Ankerite [Ca(Fe^2+^,Mg)(CO_3_)_2_]. Ankerite occurred in both reservoir and caprock proxies. In R1–R3, pre-exposure values ranged from 4.40 wt.% to 5.49 wt.%, decreasing post-exposure to 4.08–4.33 wt.%. In D1, it was not detected pre-exposure and remained absent post-exposure. In D2, it decreased from 4.02 wt.% to 3.55 wt.%. Morphologies were retained but with localized surface dulling near grain boundaries.
- Wollastonite (CaSiO_3_). Wollastonite was absent prior to exposure and formed in all samples post-exposure. In R1–R3, abundances were 0.67 wt.%, 1.08 wt.%, and 1.02 wt.%, respectively. In D1 and D2, wollastonite was recorded at 0.56 wt.% and 0.93 wt.%. It appeared as fibrous or acicular precipitates localized around sites of prior carbonate dissolution.
- Albite (NaAlSi_3_O_8_). Albite was present in every sample, and its presence in R1 had minimal increase from 4.88 to 5.75 wt.% and in R3 it was a negligible change from 5.96 wt.% preexposure to 5.94 wt.% post-exposure. In D1 and D2, albite changed from 4.71 wt.% and 4.82 wt.% to 4.93 wt.% and 4.65 wt.%, respectively, all of which are within experimental error limits due to sample heterogeneity. Grains retained sharp outlines and showed no signs of dissolution.
- Pyrite (FeS_2_). Pyrite was present across all facies. In reservoirs, pre-exposure values ranged from 4.88 wt.% (R1) to 5.87 wt.% (R2), declining to 4.67–5.41 wt.% post-exposure. In D1 and D2, pyrite decreased from 6.28 wt.% and 6.26 to 5.56 wt.% and 4.84 wt.%, respectively. SEM showed edge diffusion and oxidation halos near OM and clay interfaces in caprock samples.
- Jarosite [KFe_3_(SO_4_)_2_(OH)_6_]. No jarosite was detected in Caney shale prior to exposure to scCO_2_, in this study it was identified post-exposure in R1 (0.58 wt.%), D1 (1.24 wt.%), and D2 (1.49 wt.%), with trace detection in R2. No formation was observed in R2. It formed as fine-grained spikelet aggregates, frequently bordering anhydrite, pyrite and organic-rich regions.
- Anhydrite (CaSO_4_·2H_2_O). Anhydrite was detected pre-exposure and post-exposure in reservoir and caprock proxies; notably: 0.80 wt.% in D1 and 1.07 wt.% in D2. It appeared as thin, patchy coatings at mineral boundaries. The CaSO_4_ phase was identified as anhydrite, based on its dehydrated state and the high-temperature SEM-EDS preparation conditions. Given that EDS does not detect hydration state, gypsum or basanite cannot be ruled out entirely, but the thermal and vacuum conditions favor the anhydrite form.
- Organic Matter (CxHyOz). Organic matter was found in all samples, increasing post-exposure in every case. In R1–R3, OM increased from 5.17 wt.%, 6.57 wt.%, and 5.99 wt.% pre-exposure to 10.79 wt.%, 12.77 wt.%, and 11.89 wt.%, respectively. In D1 and D2, OM rose from 6.66 wt.% and 6.99 to 12.15 wt.% and 14.19 wt.%, respectively. Post-exposure OM showed increased surface roughness, irregularity, and porosity development.

Table 2 below summarizes the average elemental composition of each phase as detected and analyzed using SEM-EDS. Mineral phases identified via SEM-EDS were classified based on dominant elemental signatures. We acknowledge natural phases may form solid solutions; names reflect dominant composition rather than pure endmembers.

### 3.2. Chemical Elemental Mobilization

Variations in K^+^, Ca^2+^, Mg^2+^, Na^+^, SO_2_^4−^, and Fe^2+/3+^ are recorded across R1. K^+^ and Na^+^ signals decrease alongside a reduction in K-feldspar abundance (13.6 to 10.2 wt.%) and Albite (5.0 to 3.4 wt.%). Ca^2+^ and Mg^2+^ shifts correspond to marked declines in Calcite (18.0 to 11.2 wt.%) and Ankerite (5.0 to 2.1 wt.%). SO_4_^2−^ release is supported by the disappearance of Anhydrite (2.5 wt.% pre-exposure). A drop in Pyrite content (3.2 to 1.4 wt.%) aligns with trace-level Fe^2+^ observations. Illite (27 wt.%) and Quartz (24.7 wt.%) show no significant quantitative change. Minor components such as Kaolinite (<1.5 wt.%) remain within detection limits without notable change.

Ionic reductions are inferred for K^+^, Ca^2+^, Mg^2+^, SO_4_^2−^, and Fe^2+^ in D1. These align with the measured loss in K-feldspar (14.3 to 9.8 wt.%) and Illite (26.7 to 22.9 wt.%), as well as Calcite (15.4 to 9.3 wt.%) and Dolomite (12.2 to 8.0 wt.%). Sulfur-bearing phases show change, with Anhydrite declining from 3.0 to <1.0 wt.%. Pyrite decreases to 2.1 wt.%, and minor Fe signals are present in adjacent altered zones. Na^+^ remains stable, with Albite retaining 3.2 wt.%. Quartz (24 wt.%) and minor phases such as Kaolinite and organic matter (1 wt.%) do not register significant variation.

In R2, changes in K^+^, Ca^2+^, Mg^2+^, SO_4_^2−^, and Fe^2+^ are evident. K-feldspar content declines from 12.7 to 8.4 wt.%, and Illite from 21.9 to 19.2 wt.%. Calcite and Dolomite decrease to 10.1 and 6.3 wt.%, respectively, reflecting a reduction in Ca^2+^ and Mg^2+^ during carbonates reprecipitation. Anhydrite, originally present at 2.8 wt.%, is no longer detected post-exposure, consistent with increased SO_4_^2−^ signal. Pyrite falls to 2.3 wt.%, with Fe^2+/3+^ signal diffusion around previous grain boundaries. Albite (3 wt.%) and Na^+^ levels remain stable. Quartz holds at 24.6 wt.%. Kaolinite (1.1 wt.%) and organic matter (0.9 wt.%) are preserved without apparent shifts.

The reduction of Ca^2+^, Mg^2+^, K^+^, and SO_4_^2−^ is confirmed across D2. This corresponds with decreased proportions of Calcite (17.5 to 11.6 wt.%), Dolomite (11.8 to 7.5 wt.%), and K-feldspar (13.2 to 8.9 wt.%). Illite shifts slightly (19.6 to 17.1 wt.%), while Anhydrite (2.6 wt.%) is fully absent post-exposure, with elevated sulfur signals noted in former anhydrite-bearing regions. Pyrite declines to 1.7 wt.%, with minor Fe presence. Albite ranges from 3.1 to 2.6 wt.%, with negligible Na^+^ signal deviation. Quartz (~23.9 wt.%) and minor minerals such as Kaolinite, organic matter, and trace phosphates show no measurable alteration.

**Figure 6:**
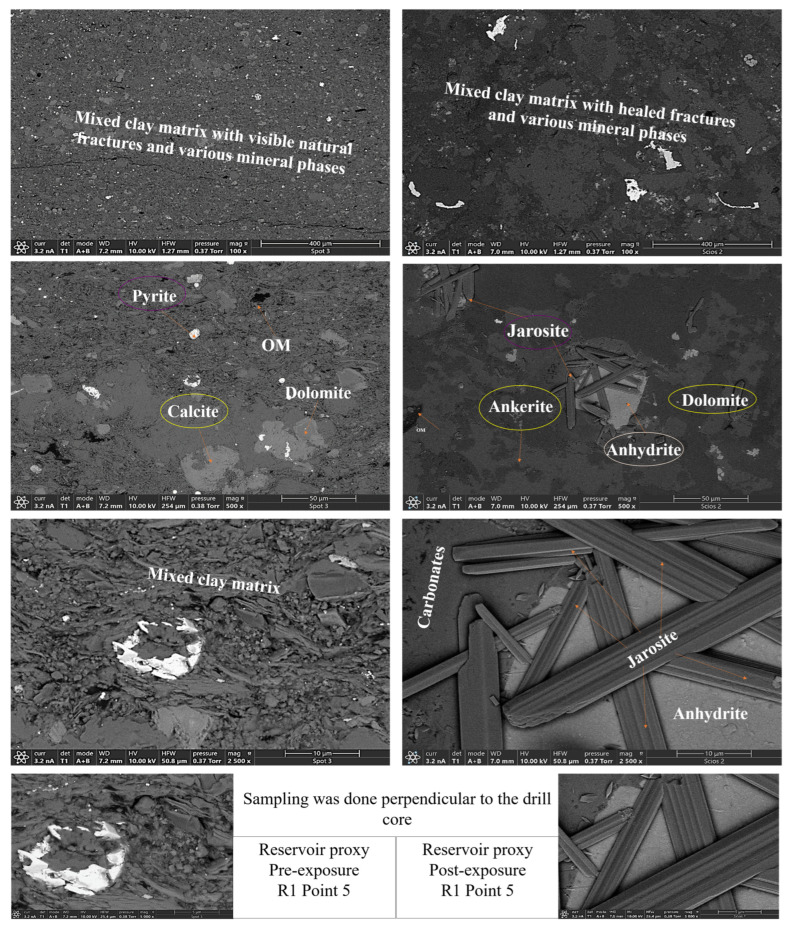
SEM of R1–Point 5 (reservoir proxy) before (**left**) and after (**right**) scCO_2_ exposure, showing mineralogical transformations. Pre-exposure images reveal calcite, dolomite, pyrite, and organic matter within a silicate framework. Post-exposure, new jarosite forms alongside dolomite, ankerite, and anhydrite near OM-rich zones. Jarosite, resulting from pyrite oxidation, enhances CO_2_ trapping through sulfate crystallization, introduces micro-porosity, and contributes to grain-scale stability, highlighting its key role in long-term sequestration and reservoir reinforcement.

**Figure 7:**
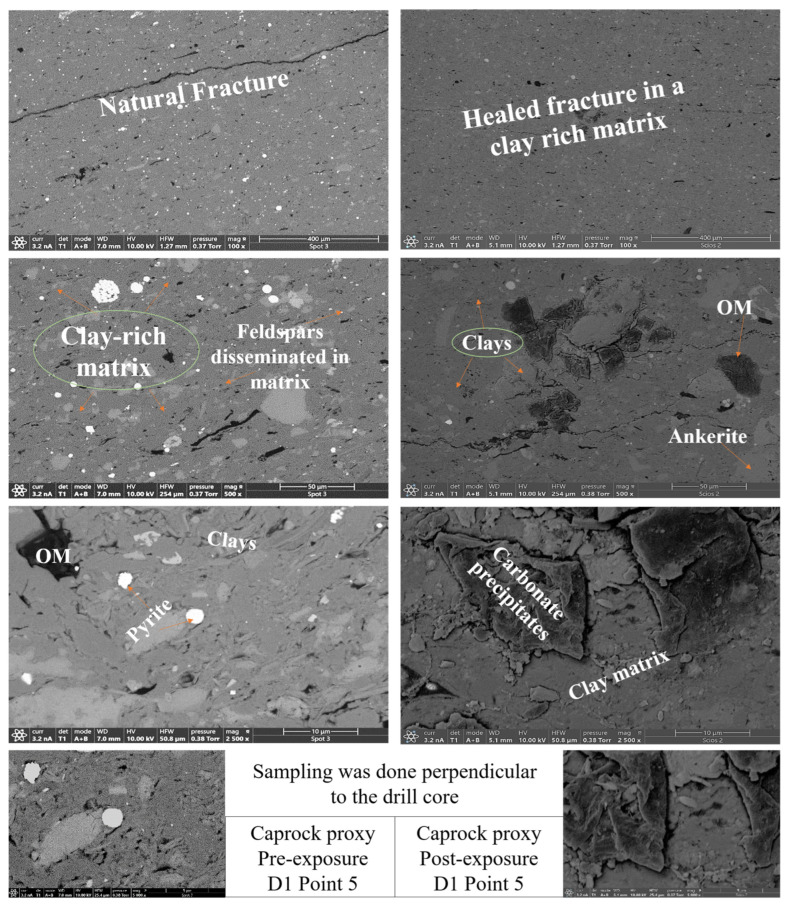
D1 Caprock proxy pre and post exposure. SEM images of D1A–Point 5 (caprock proxy) before (**left**) and after (**right**) scCO_2_ exposure, showing textural and mineralogical changes. Pre-exposure images reveal a fine-grained, clay-rich matrix with disseminated feldspars. Post-exposure, the caprock surface shows enhanced expression of clays and feldspars, alongside preserved organic matter and ankerite. The increased concentration of clays suggests mineral stabilization and enrichment, reinforcing the caprock’s sealing capacity and long-term geochemical integrity following scCO_2_ injection.

SEM-EDS elemental mapping indicates selective post-exposure mobilization of K^+^, Ca^2+^, Mg^2+^, Fe^2+^, and SO_4_^2−^ across facies. Carbonate-bearing zones in R1, R2, and R3 show marked Ca^2+^ and Mg^2+^ depletion, consistent with mineral phase volume loss from Calcite, Dolomite, and Ankerite. Pyrite-associated Fe^2+/3+^ and S(SO_4_^2−^) signals decrease, with partial redistribution observed in caprock proxies such as D2. Illite grains exhibit localized leaching of K^+^, Mg^2+^, and Fe^2+/3+^, particularly in the reservoir proxies. In contrast, Si and Al remain stable, reflecting Quartz and K-feldspar persistence. Organic matter becomes more spatially distributed post-exposure, especially along former carbonate-clay boundaries. No secondary precipitates were detected within the 30-day interval, though surface alterations indicate potential reactivity under extended conditions. Figure 6 and Figure 7 give a sample observation of a key feature observed post reaction for a caprock and reservoir proxy.

Table 3 is a summary of the ionic and elemental species mobilized during the reaction, these species are as detected through EDS. Equally, the table provides a link to the geochemical reaction paths attributed to these mobilizations.

## 4. Discussion

### 4.1. Mineral Stability and Reactivity

The early-stage mineralogical and microstructural changes observed in the Caney Shale samples have important impact on the long-term safety and effectiveness of geologic carbon sequestration. The Caney Shale demonstrates that supercritical CO_2_ (scCO_2_) exposure initiates diverse mineralogical transformations, largely governed by facies-level differences in composition, porosity, and reactivity. While the broad categories of carbonate dissolution and clay interaction have been widely reported in other shales, this study reveals the importance of sulfate-related transformations and the catalytic role of organic matter. These findings support a nuanced understanding of geochemical reactivity across mechanically distinct lithologies [15] and provide some insights for carbon storage safety and long-term relevance.

#### 4.1.1. Carbonate Phases

**Figure 8:**
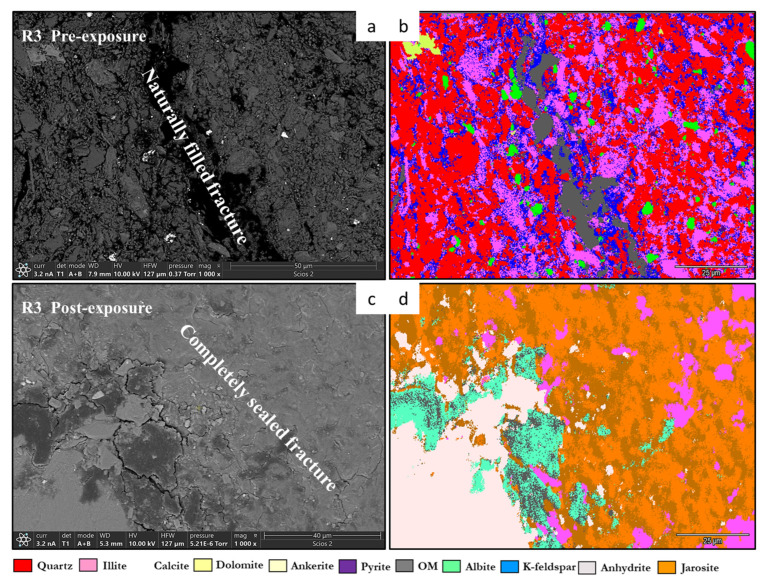
Mineral phase maps and backscattered SEM images from R3 illustrating carbonate phase evolution before and after scCO_2_ exposure. In the pre-exposure image (**a**), a naturally filled fracture is observed cutting through the matrix, with the corresponding EDS phase map (**b**) showing ankerite and calcite phases localized along the fracture and at feldspar–carbonate interfaces. Post-exposure imaging (**c**) reveals a completely sealed fracture, consistent with carbonate re-precipitation and in situ sealing. The post-exposure phase map (**d**) highlights redistribution and partial reduction of carbonate phases, with transformation at mineral boundaries and localized infill zones dominated by fine-grained reaction products. This supports evidence for carbonate re-precipitation and sealing under dry scCO_2_ conditions.

Reservoir facies such as R2A and R3A displayed extensive dissolution of calcite and ankerite. This pattern confirms earlier observations of carbonate reactivity under CO_2_-rich conditions [23], where the release of Ca^2+^, Mg^2+^, and Fe^2+^ ions initiates buffering and supports the early stages of mineral trapping [34]. In contrast to previous findings where reprecipitation was considered minimal without brine [14], this study provides evidence of secondary precipitation even under nominally dry conditions. Carbonate reactivity is a major gateway to geochemical sequestration of CO_2_. No discrete Ca-silicate phases such as wollastonite were identified. Ca–Si signals observed in Raman and EDS maps are likely attributable to mixed silicate-carbonate domains or poorly crystalline components associated with clay-carbonate interfaces [35]. Figure 8 is an illustration of these. The formation of calcium silicate (wollastonite) indicates that these carbonates did not simply dissolve but instead facilitated new stable mineral assemblages. While carbonate dissolution may initially release CO_2_ or buffering cations (e.g., Ca^2+^, Mg^2+^), subsequent carbonation and mineral re-precipitation contribute significantly to long-term geochemical trapping [13]. This dual behavior positions carbonate reactivity as a key mechanism in CO_2_ sequestration under dry supercritical conditions [36]. Such transformations are reported to arise through coupled reactions involving silicate-structural and carbonate-derived cations [32]. Their spatial localization near feldspar-carbonate-OM boundaries suggests that geochemical trapping in a mineralogically diverse shale rocks is interface-driven rather than single mineral surface reaction phenomena (homogeneous). This behavior underscores the importance of interfacial mineralogy in shaping the spatial pattern of storage efficiency.

#### 4.1.2. Clays and Feldspars

**Figure 9:**
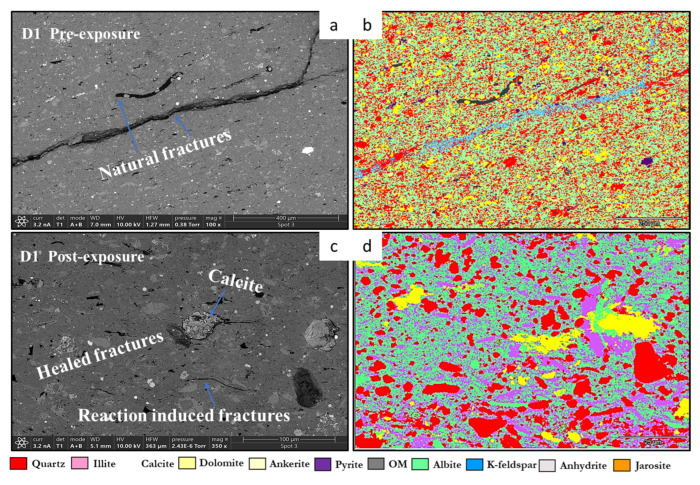
Pre- and post-exposure SEM and EDS analyses of D1 caprock facies (**a**–**d**) illustrate fracture evolution and mineral transformation. In the pre-exposure SEM image (**a**), natural fractures follow mineral boundaries, likely resulting from depositional compaction and early diagenesis. The corresponding EDS map (**b**) highlights clay-rich zones along these fractures, marking sites of mechanical weakness. Post-exposure SEM imaging (**c**) reveals calcite-filled healed fractures and newly formed reaction-induced fractures attributed to mineral dissolution and stress redistribution under scCO_2_ exposure. The EDS phase map (**d**) shows spatial redistribution of phases, with carbonate precipitation localized at fracture tips and clay–mineral interfaces. Illite edges show thinning, paragonite appears along fracture margins, and feldspar grains exhibit incipient etching. Fracture infill is composed of fine-grained alumino-silicates, consistent with in situ transformation products.

Clay minerals exhibited distinct responses across facies. In the ductile caprock settings of D1 and D2, illite displayed edge thinning alongside localized transformation into paragonite, accompanied by an overall increase in illite presence. This trend is consistent with illitization, potentially driven by minor Na^+^ availability from albite or residual interstitial fluids [37,38]. Alterations observed near clay boundaries under low-water conditions suggest that clay phases can undergo mineralogical adjustment, contrary to prior assumptions of their inert behavior in scCO_2_ environments, particularly where thin water films persist [39,40]. The increased clay content, along with progressive illitization, is associated with enhanced ductility and greater sealing capacity in the caprock proxies [30,32]. Feldspars, while structurally preserved, show signs of active chemical exchange. Slight K^+^ depletion and surface etching, particularly in R1 and R3, point to early-stage hydrolysis [41]. These reactions may facilitate the formation of secondary clays or contribute mobile cations relevant to CO_2_ trapping processes. The involvement of feldspars in such geochemical interactions reflects long-term alteration patterns observed in sedimentary basins [14], although their direct contribution to mineral trapping remains limited. Clays and feldspar serve as alteration controllers and ion exchange sites. Figure 9 illustrates the clay mobilization in the caprock proxy D1.

#### 4.1.3. Sulfide Oxidation and Sulfate Reaction Pathways

**Figure 10:**
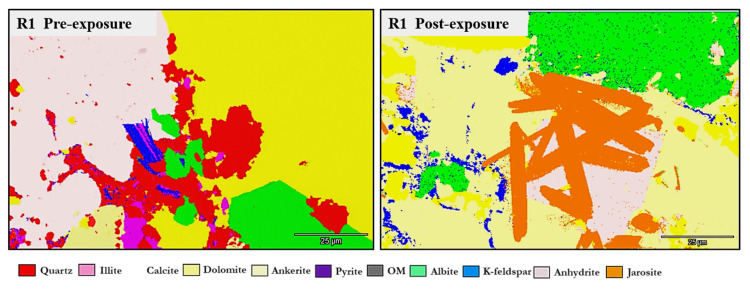
Post-exposure mineral map shows newly formed jarosite in reservoir facies with adjacent porosity development, suggesting sulfate phase persistence under localized oxidative conditions.

Formation of jarosite and additional anhydrite (CaSO_4_) was observed in both reservoir and caprock facies following scCO_2_ exposure. These phases are associated with pyrite oxidation and redistribution of sulfate from pre-existing anhydrite. Their presence in a closed system, containing only scCO_2_ and no added SO_2_, indicates that sulfate-bearing phases can form under localized oxidative conditions. Co-occurrence with organic matter and likely trace water suggests that redox activity can occur in the absence of externally supplied oxidants, supporting Fe^2+^ and SO_4_^2−^ mobility in the various facies [15]. Sulfate-bearing phases, although less frequently emphasized in shale carbon storage contexts, are observed to persist under the experimental conditions. Jarosite was identified in multiple points, and both jarosite and anhydrite are known to exhibit stability under low pH and moderate temperature [23]. Jarosite spikelets as seen in Figure 6, show vast porosity created, giving more room for sequestration. Figure 10 shows the pre and postexposure EDS of the jarosite linked spot. Here, the reaction is hypothesized to initiate through pyrite oxidation, likely accelerated by the presence of trace porewater and redox-active OM [34]. This process releases Fe^2+^ and S^2−^, which, under oxidizing conditions, transition into Fe^3+^ and SO_4_^2−^ [42], ultimately forming jarosite through a precipitation reaction:

(1) $$K^{+}(aq)+3{Fe}^{3+}(aq)+2{SO}_{4}^{2-}(aq)+6H_{2}O\to K{Fe}_{3}{\left({SO}_{4}\right)}_{2}{\left(OH\right)}_{6}(s)+6H^{+}(aq)$$

Equation (1) shows the jarosite formation reaction which is thermodynamically favored under acidic, low ionic strength conditions [13]. Its appearance in a brine-free, scCO_2_ setting underscores the capability of shale-hosted systems to support complex redox mineral transformations without exogenous oxidants. Our results indicate that sulfate mineral formation can occur alongside carbonate reactivity, particularly in organic-rich zones where sulfide-CO_2_ interactions are spatially linked.

#### 4.1.4. Organic Matter: A Chemically Active Interface

**Figure 11:**
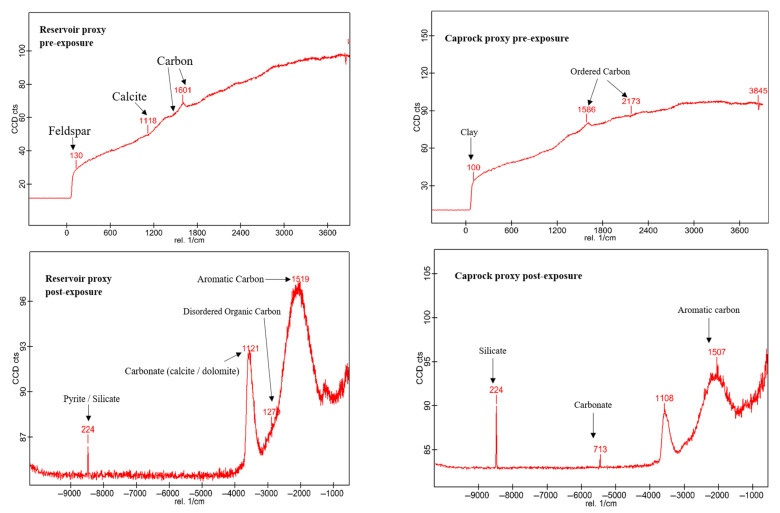
Raman spectra of reservoir (R1) and caprock (D2) facies before and after exposure to scCO_2_ at 60 °C and 17.8 MPa. In the reservoir proxy (R1), carbon signals evolve from a broad disordered peak (~1601 cm^−1^) to distinct D and G bands at 1279 cm^−1^ (disordered/sp^3^ carbon) and 1519 cm^−1^ (aromatic/sp^2^ carbon), indicating increased structural ordering and aromatization. In the caprock proxy (D2), a similar transformation is observed, with the pre-exposure band at 1586 cm^−1^ shifting to a more defined aromatic signal at 1507 cm^−1^ post-exposure. Peaks associated with carbonate (~1108–1121 cm^−1^), silicate (~224 cm^−1^), and clay (~100–130 cm^−1^) are also present. These spectra confirm the retention and transformation of organic matter toward more graphitized forms under scCO_2_ conditions.

Organic matter played a dual role across all facies, Figure 11 illustrates this with R1 and D2. OM acted both as a high-affinity sorbent for scCO_2_ and as a catalytic interface for redox-mediated mineral reactions [43]. In ductile caprock facies, organic surfaces underwent fragmentation and porosity development, which enhanced their ability to store scCO_2_ in micropores [25]. These changes also supported electron transfer reactions, especially near pyrite and clay edges, fostering the nucleation of new mineral phases [44]. Organic matter can be defined in our context as a chemically active interface. The formation of illite-OM aggregates and secondary mineral patches at OM-clay boundaries confirm that organic matter supports not only CO_2_ uptake but also chemical transformation. Previous studies have shown the sorptive value of OM [12], but the current results demonstrate its capacity to induce mineral precipitation and stabilize reaction fronts. This behavior extends the functional relevance of organic matter from passive retention to active geochemical regulation within shale-hosted sequestration systems.

### 4.2. Relevance for Geochemical Sequestration

The mineral transformations and reactivity patterns observed in this study suggest that Caney Shale facies contribute to CO_2_ storage in distinct yet complementary ways. Reservoir units serve as the primary trapping domains through carbonate dissolution and re-precipitation. Ductile caprock facies, on the other hand, operate as chemical seals, limiting vertical migration and stabilizing CO_2_ through redox and sulfate-based reactions.

#### 4.2.1. Reservoir Proxies

Proxies R1 through R3 are characterized by high quartz content combined with moderate amounts of reactive feldspars, carbonates, clays, and organic matter. Spatially localized dissolution and precipitation were observed, primarily concentrated along mineral boundaries and microstructural interfaces. R2 exhibited the most extensive carbonate loss, particularly of ankerite, followed by irregular precipitation of both carbonate and silicate phases. R3, with lower carbonate content, displayed greater expression of organic matter and sulfate reactivity, indicating multiple coexisting trapping mechanisms [45]. Such mineral–fluid interactions align with findings in other mixed-lithology reservoirs, where uneven carbonate distribution contributes significantly to mineralogical trapping [34]. The detection of Ca–Si-rich areas at silicate–carbonate-OM interfaces suggests the formation of calcium-bearing silicate assemblies [43]. While wollastonite (CaSiO_3_) was not conclusively identified, Raman and EDS data reveal spatially correlated Ca^2+^ and SiO_2_ signals consistent with possible low-crystallinity or transitional Ca–silicate compounds [44]. These mineral associations, though unresolved in structure, may represent additional trapping phases not typically emphasized in standard CCS models [7,46]. Such variability in mineral phases supports localized reactivity that could potentially enhances CO_2_ retention in texturally heterogeneous zones. The reservoir proxies are thus accounted for reactive porosity and multiphase trapping.

#### 4.2.2. Caprock Proxies

Caprock proxies D1 and D2 retained structural integrity and low porosity following scCO_2_ exposure. Their composition rich in clays and organic matter, and the emergence of sealing-associated phases suggest a dominant geochemical sealing role rather than bulk-phase CO_2_ mineralization [47]. Compact microtextures were preserved post-exposure, and the presence of sulfate and redox-active phases was confirmed by both SEM-EDS and Raman analyses [3,48]. These features are consistent with current caprock performance models that emphasize ion retention and interfacial buffering over mechanical deformation. Sulfate mineralization and redox processes involving organic matter occur in the absence of external oxidants or brine immersion. The sealing behavior is reinforced by microscale dissolution–precipitation reactions that operate across reactive boundaries and enhance caprock resilience without compromising structural integrity [49]. These processes are not just passive but actively reinforce sealing through coupled dissolution and precipitation at the microscale. Caprock proxies are thus accounted for ensuring sealing (geochemically proven), hence confirming the dual nature of shales as for CO_2_ containment and leak prevention.

#### 4.2.3. Integrated Storage Performance and Relevance for CCS Design

Facies-dependent responses in Caney Shale illustrate the function of an integrated trapping system in which porosity access, geochemical transformation, and redox buffering act concurrently. Reservoir zones enable CO_2_ migration and support mineral conversion along carbonate and sulfate pathways. Caprock layers contribute by limiting leakage through chemical immobilization and formation of clay barrier phases at reactive interfaces. Formation of sulfate-bearing minerals, calcium–silicate domains, and clay–organic composites under scCO_2_ exposure broadens the range of sequestration pathways beyond traditional carbonate-based considerations.

SEM-EDS analyses of Caney Shale samples exposed to scCO_2_ reveal dual effects: local mineral transformations support both self-sealing and mechanical weakening processes. Carbonate dissolution was frequently accompanied by precipitation of secondary phases at grain boundaries and within pore spaces, especially in reservoir facies such as R2 and R3. These fine-scale precipitates suggest activation of self-sealing pathways, even without brine, aligning with prior findings in clay–carbonate systems [35].

Similarly, microstructural weakening was evident in caprock facies. Illite exhibited sheet-edge thinning and minor delamination; pyrite showed early oxidation; and organic matter displayed fragmentation with increased surface porosity. Such alterations, most prominent in D1 and D2, raise concerns about clay integrity under prolonged exposure, consistent with reported CO_2_-induced softening of sealing units under low-water conditions [3]. The interplay between sealing and weakening is spatially variable and phase-dependent. In carbonate-rich zones, re-precipitation may enhance capillary sealing. In phyllosilicate-dominated domains, structural degradation may reduce long-term integrity, particularly under stress cycles associated with injection operations [2,28,50]. Observed phase transformations support a wider trapping framework that incorporates both primary reactions and secondary mineral development without reliance on brine or supplemental oxidants. Caney Shale demonstrates geochemical and structural reliability for long-term CO_2_ retention, with distinct yet complementary contributions from reservoir and caprock settings. Table 4 provides an overview of literature with the common mineral phases associated with CCUS and how they fit the purpose of our research.

### 4.3. Geochemical Insights

**Figure 12:**
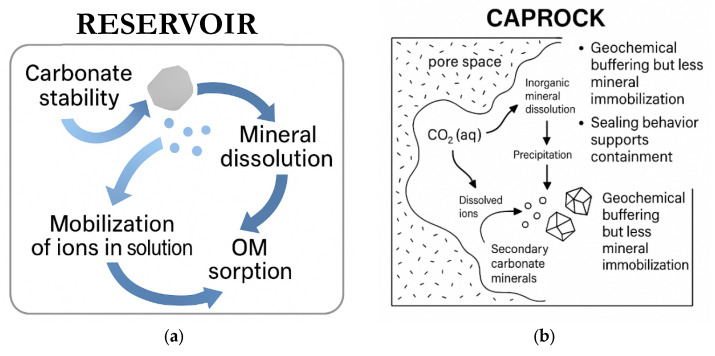
Shale exhibits dual-functionality for CO_2_ storage governed by facies-specific geochemical behavior. (**a**) The reservoir acts as a reactive matrix, promoting mineral dissolution, elemental redistribution, and stable secondary phase precipitation essential for geochemical trapping. (**b**) The caprock maintains low reactivity, enabling structural preservation through limited transformation and clay-stabilization, thereby supporting long-term seal integrity.

Mineral reactivity in the Caney Shale under scCO_2_ exposure is highly localized, driven by the spatial arrangement of reactive phases and interfacial microenvironments. Transformations occurred not uniformly, but along grain boundaries where carbonates, clays, sulfides, and organic matter converge. These interfaces enabled coupled processes such as ion exchange, redox cycling, and secondary phase nucleation. Consequently, this extends trapping pathways beyond carbonate dissolution. In Figure 12, illustrations of the observed mineral evolution reflect not just composition, but the connectivity and proximity of reactive constituents in both facies. Organic matter enhanced reactivity by supporting electron transfer and acting as a nucleation site, while stable phases like quartz and feldspar constrained reactions spatially. Overall, trapping efficiency in the Caney Shale appears controlled less by bulk mineralogy than by the distribution and interaction of components at the microscale.

## 5. Conclusions

Exposure of Caney Shale to pure scCO_2_ at 60 °C and 17.23 MPa (2500 psia) induces rapid, phase-specific mineral reactions relevant to long-term carbon storage. Quartz remains chemically inert, while carbonates dissolve and locally re-precipitate as calcium- and iron-rich secondary phases. Illite exhibits edge alteration and cation leaching; pyrite undergoes early-stage oxidation; and organic matter becomes more porous, supporting redox activity at mineral interfaces. SEM-EDS analysis confirms that mineral trapping initiates under dry conditions, with nanometer-scale precipitates forming at grain boundaries and pore surfaces. Reactions are not uniformly distributed but are instead governed by spatial variability in mineral associations, surface properties, and interface geochemistry. Insights gained from this experiment include the following:

- Localized porosity development enhances CO_2_ injectivity, while secondary mineral precipitation at grain contacts and pore throats contributes to self-sealing behavior, supporting containment stability.
- Demonstrated mineral trapping in dry scCO_2_ (no added brines) systems confirms that water is not a prerequisite for initiating geochemical containment, with in situ precipitation providing a viable mechanism for immobilizing (sequestering) injected CO_2_.
- Facies-dependent reactivity, mineral phase and ionic species distribution support a naturally evolving balance between fluid migration pathways and geochemical seals. This allows reactive zones (reservoirs) to co-exist with stable, low-permeability zones (caprocks).
- Existing shale development from hydraulic fracturing offers an operational advantage, enabling CO_2_ storage to leverage established well infrastructure, reservoir access strategies, and field-scale monitoring systems.

Future work should explore sulfate phase formation, including jarosite (KFe_3_(OH)_6_(SO_4_)_2_), using combined with reactive-transport modeling and calculations. Integrating EDS-based stoichiometry with kinetic datasets will help quantify CO_2_ uptake across key mineral phases. Extending experiments to other shales will provide a crosslink benchmarking. Incorporating mineral-scale reaction rates into geomechanical and geophysical models can improve leakage-risk assessments, while EBSD and FIB-SEM will enable deeper characterization of reaction zones and deformation below the surface. These steps will sharpen monitoring strategies, and ultimately strengthen confidence in shale-dominated formations as long-term, self-adjusting CO_2_ sequesters.

## Figures and Tables

**Table 1:** Literature review and gaps identified for a refined approach to our methodology.

Author(s)	Focus	Research Gaps
[17,18]	Numerical Simulations of CO_2_ in Geological Settings	Limited empirical data on physicochemical interactions at the mineralogical level in shales. Need for experimental validation of simulated predictions and theoretical models.
[19,20]	Geologic Carbon Sequestration Review	High costs and energy requirements for CO_2_ capture; need for cost reduction and efficiency enhancement.
[3,5,17,21,22]	Caprock Integrity and Fracture Dynamics	Need for long-term studies need to understand the evolution of fissures under continuous CO_2_ flow. The importance of considering hydrological factors in geological stability assessments.
[2,23,24,25]	Pore Structure Alterations	Microscale and nanoscale analysis, shale-specific studies, and controlled experiments are vital to assess structural changes and ensure long-term CO_2_ storage integrity.
[5,7,14,26,27]	Subcritical and Supercritical CO_2_ Effects on Shale	Robust simulations and further studies are essential to understand shale sensitivity to CO_2_ under varying conditions and optimize EOR strategies.
[28,29,30,31]	CO_2_ Storage Capacity and Monitoring	Targeted modeling, localized studies, and field validation are essential to predict CO_2_–shale interactions, refine capacity estimates, and assess long-term storage risks.
[5,15,26,27,32,33]	Impact of CO_2_—Rock Interactions	Comprehensive experimental and modeling studies are needed to understand shale reactivity, nanoconfinement, water-chemistry interactions, and long-term CO_2_ impacts across diverse geological settings.

**Table 2:** Average EDS classification of mineral phases.

Phase	Main Elements (wt.%)
Quartz	Si (34.5), O (49.9), Fe (6.0), C (3.9), Al (2.9)
Organic Matter (OM)	C (43.0), O (34.5), Si (16.6), Ca (2.6), Al (2.0)
Calcite	Ca (21.3), O (52.0), Si (16.3), C (6.3), Al (2.0)
Pyrite	Fe (35.8), S (24.1), O (20.8), Si (12.0), C (3.2)
Illite	Si (26.6), O (50.9), Al (10.0), K (3.5), Fe (3.0)
Dolomite	Ca (31.5), Mg (20.4), O (45.1), C (2.1)
Kaolinite	Si (29.5), O (52.7), Al (9.0), C (3.7), Fe (1.5)
Paragonite	Na (4.5), Al (12.6), Si (31.0), O (48.0), Fe (1.2)
Wollastonite	Ca (22.0), Si (28.5), O (44.0), C (3.5), minor Al
Ankerite	Ca (22.5), Fe (12.4), Mg (10.2), C (5.8), O (49.1)
Albite	Na (6.8), Al (19.1), Si (35.2), O (37.5), trace Ca
Jarosite	K (4.5), Fe (23.4), S (13.2), O (50.0), OH present
Anhydrite	Ca (26.2), S (18.0), O (55.8)

**Table 3:** From ionic mobilizations to sequestration, a summary of the potential ionic mobilizations.

Ionic Species	Primary Mineral Phase Sources	Facies Observed	Post-Exposure Observation (Quantified in wt.%)	Possible Geochemical Path
K^+^	K-feldspar, Illite	R1, R2, D1, D2, R3	K-feldspar reduction (13.6 to 7–10%); slight Illite shift	Leaching from feldspars and clay edges
Na^+^	Albite	R1, R3, D2	Minor Albite decline (5.0% to 2.6–3.4%)	Limited Na^+^ exchange
Ca^2+^	Calcite, Dolomite, Ankerite	R1, R2, R3, D1, D2	Redistribution among carbonate phases; net Ca^2+^ preserved	Partial dissolution and re-precipitation
Mg^2+^	Dolomite, Illite, Ankerite	R2, R3, D2, D1	Mg-bearing carbonates reduced; Dolomite often retained	Phase transition and reallocation
Fe^2+^/Fe^3+^	Pyrite, Illite, Ankerite	D1, D2, R2, R3	Pyrite decreased (up to 50%); Fe detected near former grains	Oxidation and surface destabilization
SO_4_^2−^	Anhydrite, Pyrite	R1, D1, D2, R2, R3	Anhydrite loss, S redistributed	Sulfate release from dissolution/oxidation
Al^3+^	K-feldspar, Albite, Illite	All facies	No significant compositional change	Structurally retained in aluminosilicates
Si^4+^	Quartz, K-feldspar, Illite, Albite	All facies	Quartz (~24–25%) stable throughout	Framework remains chemically inert
C (elemental) CO_3_^2−^	Calcite, Dolomite, Ankerite	All facies	Carbon and carbonates retained via phase shifts, not net loss	Re-precipitation or phase conversion
S (Elemental)	Pyrite, Anhydrite	D2, R2, R3	Sulfur detected post-Anhydrite; diffused spatially	Sulfate migration from sulfates/sulfides
P/PO_4_^3−^	Apatite, trace organics	D2, R3 (trace levels)	Stable in isolated inclusions	Largely inert under dry CO_2_

**Table 4:** Comparing observed mineralogical changes with CO_2_ interactions in shales.

Mineral Phase	Chemical Formula	Rationale in CCUS	Occurrence in Shales	Relevance to CCUS
Calcite	CaCO_3_	Forms during CO_2_ sequestration via reaction with calcium-bearing minerals.	Common carbonate mineral in shales.	Relevant due to carbonate precipitation under CO_2_-rich conditions.
Dolomite	CaMg(CO_3_)_2_	Forms from interactions of CO_2_ with calcium and magnesium-rich minerals.	Present in some shale formations; associated with carbonate deposits.	Plays a role in carbonate mineralization under CO_2_ sequestration.
Magnesite	MgCO_3_	Forms when CO_2_ reacts with magnesium-bearing minerals.	Rare in shales, mainly found in magnesium-rich environments.	Forms stable carbonate phases during CO_2_ sequestration.
Siderite	FeCO_3_	Iron carbonate that forms in CO_2_-rich environments.	Occasionally found in Fe-rich shales, but more common in sedimentary rocks.	Can store CO_2_ in carbonate form but limited occurrence in shales.
Quartz	SiO_2_	Stable silicate mineral in shales, largely unreactive to CO_2_.	Common silicate mineral in shales, a major constituent of sandstones.	Mechanically stable but chemically inert under CO_2_ exposure.
Illite	(K,H_3_O)(Al,Mg,Fe)_2_(Si,Al)_4_O_10_[(OH)_2_	Clay mineral influencing shale porosity and permeability under CO_2_ exposure.	Frequent in shales as a clay mineral affecting permeability.	Affects shale permeability and reactivity with CO_2_.
Montmorillonite	(Na,Ca)_0_._3_(Al,Mg)_2_Si_4_O_10_(OH)_2_·nH_2_O	Swelling clay mineral that absorbs CO_2_, altering shale properties.	Found in clay-rich shales, particularly those with high swelling potential.	Modifies pore structure and water retention upon CO_2_ exposure.
Kaolinite	Al_2_Si_2_O_5_(OH)_4_	Clay mineral with minor interactions with CO_2_.	Occurs in some shales but not a dominant mineral.	Minor role in CO_2_ reactivity, mainly affects shale composition.
Ankerite	Ca(Fe^2+^,Mg,Mn)(CO_3_)_2_	Iron and magnesium carbonate forming under CO_2_ sequestration conditions.	Found in iron-rich sedimentary formations, including some shales.	Potentially relevant for mineral trapping of CO_2_.
Chlorite	(Mg,Fe^2+^,Fe^3+^,Al)_6_(Si,Al)_4_O_10_(OH)_8_	Clay mineral influencing CO_2_-induced alterations in shales.	Occurs in some shales, affecting fluid interactions.	Affects CO_2_-rock interactions by modifying clay stability.
Pyrite	FeS_2_	Common sulfide in shales, oxidizing under CO_2_ influence.	Common in organic-rich shales, particularly those with high sulfur content.	Oxidation influences acid generation, affecting mineral trapping.
Feldspar	KAlSi_3_O_8_—NaAlSi_3_O_8_—CaAl_2_Si_2_O_8_	Silicate mineral that weathers in CO_2_ environments.	Common framework silicate mineral in various shales.	Minor role in CO_2_ sequestration; undergoes limited chemical change.
Hematite	Fe_2_O_3_	Iron oxide that forms from pyrite oxidation during CO_2_ sequestration.	Minor iron oxide phase in shales formed from oxidation processes.	May form secondary precipitates upon CO_2_ exposure.
Anhydrite	CaSO_4_	Sulfate mineral present in caprocks affecting CO_2_ storage integrity.	Common in evaporite-bearing shales and caprocks.	Contributes to caprock integrity in sequestration sites.
Anhydrite	CaSO_4_·2H_2_O	Hydrated sulfate mineral influenced by CO_2_-rich fluids.	Hydrated form of anhydrite, often found in caprocks overlying shales.	Influences CO_2_ migration in formations containing gypsum.
Halite	NaCl	Salt mineral forming low-permeability barriers in caprocks.	Evaporite mineral occasionally present in shale formations.	Enhances caprock sealing potential, reducing CO_2_ leakage.
Serpentine	(Mg,Fe)_3_Si_2_O_5_(OH)_4_	Silicate mineral reacting with CO_2_ to form magnesite.	Occurs in some altered shales with high magnesium content.	Can interact with CO_2_ under specific geochemical conditions.
Olivine	(Mg,Fe)_2_SiO_4_	Silicate mineral reacting with CO_2_ to facilitate mineral sequestration.	Found in ultramafic environments but rare in shales.	Minor direct role in CO_2_ sequestration in shales.
Plagioclase	(Na,Ca)(Si,Al)_4_O_8_	Silicate feldspar undergoing carbonation reactions with CO_2_.	Common in feldspar-rich shales and sandstones.	Participates in feldspar weathering reactions under CO_2_ influence.
Smectite	(Ca,Na)_0_._33_(Al,Mg)_2_(Si_4_O_10_)(OH)_2_·nH_2_O	Clay group mineral swells upon CO_2_ exposure, modifying rock properties.	Occurs in clay-rich shale formations, affecting fluid movement.	Clay swelling may alter CO_2_ migration pathways.
Brucite	Mg(OH)_2_	Magnesium hydroxide that reacts with CO_2_ forming magnesite.	Rare in shales but found in magnesium-rich alteration zones.	Relevant in carbonation processes for CO_2_ trapping.
Forsterite	Mg_2_SiO_4_	High-Mg silicate reacting with CO_2_ for mineral sequestration.	More common in ultramafic formations, rare in shales.	Limited relevance in shales; reacts with CO_2_ in ultramafic rocks.
Talc	Mg_3_Si_4_O_10_(OH)_2_	Magnesium silicate that alters during CO_2_ interactions.	Occurs in talc-carbonate altered zones; uncommon in shales.	Plays minor role in mineral transformations in CO_2_ storage.
Mariposite	Cr-muscovite	Chromium-bearing mica associated with carbonated ultramafic rocks.	Occasionally found in altered metamorphic environments, rare in shales.	Not directly involved in CO_2_ trapping but alters rock properties.
Fuchsite	Cr-muscovite	Green, chromium-bearing mica found in carbonated environments.	Rarely found in shales; more common in metamorphic terrains.	Limited role in CO_2_ interactions due to mineral stability.
Zeolites	Mx/n [(AlO2)x(SiO2)y] · zH2O	Adsorbs CO_2_, enhancing storage capacity in shales.	Uncommon in natural shale formations but widely used in CO_2_ capture studies.	Relevant in artificial CO_2_ capture applications but rare in shales.
Muscovite	KAl_2_(AlSi_3_O_10_)(OH)_2_	Stable mineral in shales, does not significantly react with CO_2_ under sequestration conditions.	Common in shales as a mica mineral, contributing to overall mineral composition.	Minimal role in CO_2_ sequestration due to chemical stability.
Jarosite	KFe_3_(SO_4_)_2_(OH)_6_	Forms in acidic environments and is not relevant for CO_2_ sequestration in typical shale formations.	Not common in shales; forms in oxidizing, acidic conditions, often as a sulfide weathering product.	Not relevant for CCUS in shales due to formation constraints.
Dawsonite	NaAlCO_3_(OH)_2_	Potential mineral for CO_2_ trapping in sandstone formations through carbonate precipitation.	Rare; more common in sandstone reservoirs where CO_2_ mineral trapping occurs.	Relevant in sandstone-hosted sequestration but not typically found in shale settings.

## Data Availability

The original contributions presented in this study are included in the article. Further inquiries can be directed to the corresponding authors.
